# Congenital heart defects: familial recurrence patterns in Sweden

**DOI:** 10.1093/eurheartj/ehaf1048

**Published:** 2026-01-08

**Authors:** Kalliopi Kazamia, Sara Ekberg, Caroline E Dietrich, Håkan Eliasson, Marie Wahren-Herlenius, Gunnar Bergman

**Affiliations:** Department of Pediatric Cardiology, Stockholm-Uppsala, Karolinska University Hospital, Eugeniavägen 23, C8:34, Solna, Stockholm, Sweden; Department of Women’s and Children’s Health, Karolinska Institutet, Stockholm, Sweden; Red Door Analytics AB, Stockholm Sweden; Department of Medicine Solna, Clinical Epidemiology Division, Karolinska Institutet, Stockholm, Sweden; Department of Medicine Solna, Clinical Epidemiology Division, Karolinska Institutet, Stockholm, Sweden; Department of Pediatric Cardiology, Stockholm-Uppsala, Karolinska University Hospital, Eugeniavägen 23, C8:34, Solna, Stockholm, Sweden; Department of Women’s and Children’s Health, Karolinska Institutet, Stockholm, Sweden; Division of Rheumatology, Department of Medicine, Karolinska Institutet, Stockholm, Sweden; Broegelmann Research Laboratory, Department of Clinical Science, University of Bergen, Bergen, Norway; Department of Pediatric Cardiology, Stockholm-Uppsala, Karolinska University Hospital, Eugeniavägen 23, C8:34, Solna, Stockholm, Sweden; Department of Women’s and Children’s Health, Karolinska Institutet, Stockholm, Sweden

**Keywords:** Congenital heart disease, Familial recurrence, Population-based register, Maternal comorbidities

## Abstract

**Background and Aims:**

Congenital heart defects (CHD) aggregate in families, but recurrence patterns across kinships and generations remain incompletely understood. In light of improved survival and diagnostic precision, updated population-based estimates are needed. This study aimed to investigate familial recurrence patterns of CHD among relatives using nationwide Swedish register data.

**Methods:**

A retrospective, population-based case-control study was conducted, including 51 778 individuals with CHD born between 1987 and 2017 and 522 543 matched controls. Relatives (parents, full siblings, half-siblings, and offspring) were identified through linkage to national health and population registers. Logistic regression with robust standard errors clustered on maternal ID was used to estimate odds ratios (ORs) and 95% confidence intervals (CIs). Dose–response relationships, kinship-specific associations, and interactions with maternal comorbidities (diabetes, hypertension, and obesity) were explored.

**Results:**

Among individuals with at least one affected relative, the OR for CHD was 2.71 (95% CI 2.60–2.83), increasing with each additional affected relative (OR per relative 2.55; 95% CI 2.46–2.64). Recurrence was strongest for mothers (OR 3.12), full siblings (OR 3.22), and offspring (OR 3.18) and lower for fathers and half-siblings. A dose–response was observed by number of affected siblings and offspring. The association between maternal CHD and CHD in index individuals was not explained by maternal comorbidities.

**Conclusions:**

Congenital heart defect in a relative (parent, full or half-siblings, or offspring) is associated with CHD in the index individual, with recurrence patterns varying by kinship and number of affected relatives. These findings may inform genetic counselling and reproductive planning.

## Structured Graphical Abstract

**Structured Graphical Abstract:**
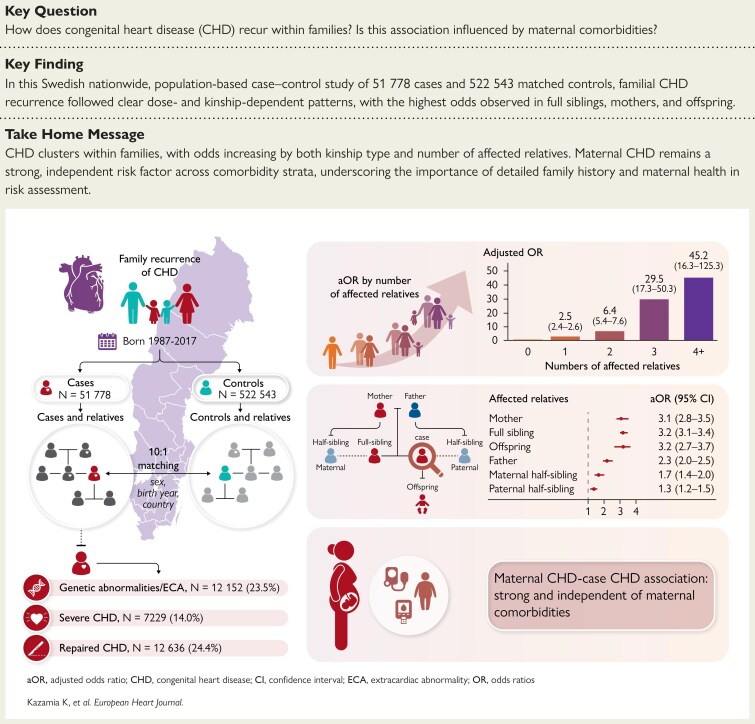
Nationwide population-based case–control study in Sweden (birth years 1987–2017) illustrating dose–response patterns by number of affected relatives and kinship-specific gradients in familial recurrence of congenital heart defects (CHD). Adjusted odds ratios from logistic regression increase with each additional affected relative and are strongest for mothers, full siblings, and offspring, with weaker associations for fathers and half-siblings. Created in BioRender. Kazamia, K. (2025) https://BioRender.com/lyukqij.

**See the editorial comment for this article ‘The conundrum of the causation of congenital heart disease’, by D.S. Celermajer, https://doi.org/10.1093/eurheartj/ehaf953.**

## Introduction

Congenital heart defects (CHD) are the most common congenital malformations, with reported birth prevalence varying worldwide depending on diagnostic practices and time periods.^1,2^ In Scandinavia, population-based studies report a birth prevalence of 13.7 per 1000 in Norway^3^ and approximately 20 per 1000 in Sweden,^4^ likely reflecting improved detection of mild lesions in more recent years. Congenital heart defects represent a broad spectrum of structural cardiac malformations, ranging from minor defects to critical conditions,^5^ requiring lifelong, highly specialized medical care.^6^

The pathogenesis of CHD is complex and multifactorial, involving both genetic and environmental factors.^7–9^ Recent genomic studies estimate that genetic contributions account for approximately 40% of cases,^10^ while maternal conditions such as diabetes, obesity, and hypertension are established risk factors.^11–15^

Although most CHD cases are considered sporadic, several large studies have demonstrated familial clustering of CHD, with higher recurrence risks among first-degree relatives (FDRs).^16–20^ The extent and patterns of this familial recurrence remain incompletely understood—especially in the context of modern diagnostic advancements and shifting prevalence trends.

Advances in paediatric cardiology and cardiac surgery have significantly improved survival, with most individuals with CHD now reaching reproductive age.^21^ This shift has led to an increasing demand for accurate, evidence-based counselling on recurrence risks in offspring. Large-scale, population-based studies are crucial for understanding familial recurrence patterns of CHD and informing genetic counselling.^22^ In this context, Scandinavian health registers provide unique research opportunities due to their nationwide coverage and capacity to link family members across generations.

This study used linked data from Sweden’s national health and population registers^23,24^ to examine the association between CHD in relatives and CHD in index individuals, assess how this association varies with the number of affected relatives, and explore kinship-specific recurrence patterns across parents, siblings, and offspring. We also explored whether maternal comorbidities such as hypertension, pregestational diabetes, and obesity modified the association between maternal CHD and CHD in index individuals. By leveraging the largest and most contemporary Scandinavian cohort to date, this study provides updated evidence on familial recurrence in the context of modern diagnostic practices and survival. These population-level data aim to inform genetic counselling and improve understanding of recurrence patterns in affected families.

## Methods

### Study design and setting

This was a Swedish population-based case-control study utilizing nationwide registers to create a multigenerational cohort, using the unique personal identification number assigned to all Swedish residents.^23^ Data were retrieved from the National Patient Register (NPR),^24^ the Medical Birth Register (MBR),^25^ the Multi-Generation Register (MGR), and the Cause of Death Register.^26^ Detailed descriptions of each data source and their coverage are provided in Supplementary data online, *Appendix A*.

This study included all eligible individuals with CHD born in Sweden between 1987 and 2017, together with matched population controls identified through nationwide registers. The sample size was therefore determined by register coverage rather than by an *a priori* power calculation. The large population-based design provided sufficient statistical precision for all planned analyses.

### Study population

Index individuals were defined as all persons with any diagnosis of CHD, recorded at any age, between 1987 and 2017 and identified in the NPR using International Classification of Diseases (ICD) versions 9 and 10 (ICD-9 and ICD-10). Congenital heart defects included all structural malformations of the heart and great vessels, including bicuspid aortic valve; full diagnostic code lists are provided in Supplementary data online, *Appendix B*.

Each index individual was matched to 10 population controls by exact year of birth (range 1987–2017), sex, and county of residence at birth.

FDRs—parents, siblings, and children—as well as half-siblings of both index individuals and controls were identified via the MGR. The resulting multigeneration cohort (index individuals, controls and their relatives) was further linked to the NPR, MBR, and Cause of Death Register to obtain data on cardiac diagnoses as well as extracardiac and genetic abnormalities, pregnancies, maternal comorbidities, care instances, and causes of death until 31 December 2020. For FDRs born before 1987, cardiac diagnoses and maternal comorbidities were identified using ICD-8 codes.

To contextualize CHD prevalence trends over time (presented in the Supplementary data, Appendix D) the total number of live births in Sweden between 1987 and 2017 (*n* = 3 214 974) was retrieved from Statistics Sweden.

### Exclusion criteria

To reduce misclassification bias, we developed a structured set of exclusion criteria based on a systematic review of diagnostic patterns in the register data. These criteria were designed to exclude physiological findings, misclassified maternal records, and isolated or implausible diagnoses unlikely to represent true CHD.

For diagnostic exclusions, index cases fulfilling the exclusion criteria were excluded together with their matched controls to maintain comparability. In contrast, relatives fulfilling the exclusion criteria were not excluded but were instead classified as unaffected. A full description of the exclusion criteria, including the rationale, diagnostic codes, and diagnosis-specific considerations (e.g. the overlap of patent foramen ovale and secundum atrial septal defect under ICD-9), is provided in Supplementary data online, *Appendix B*  *and C*, *Tables S1–S4*.

**Figure 1:**
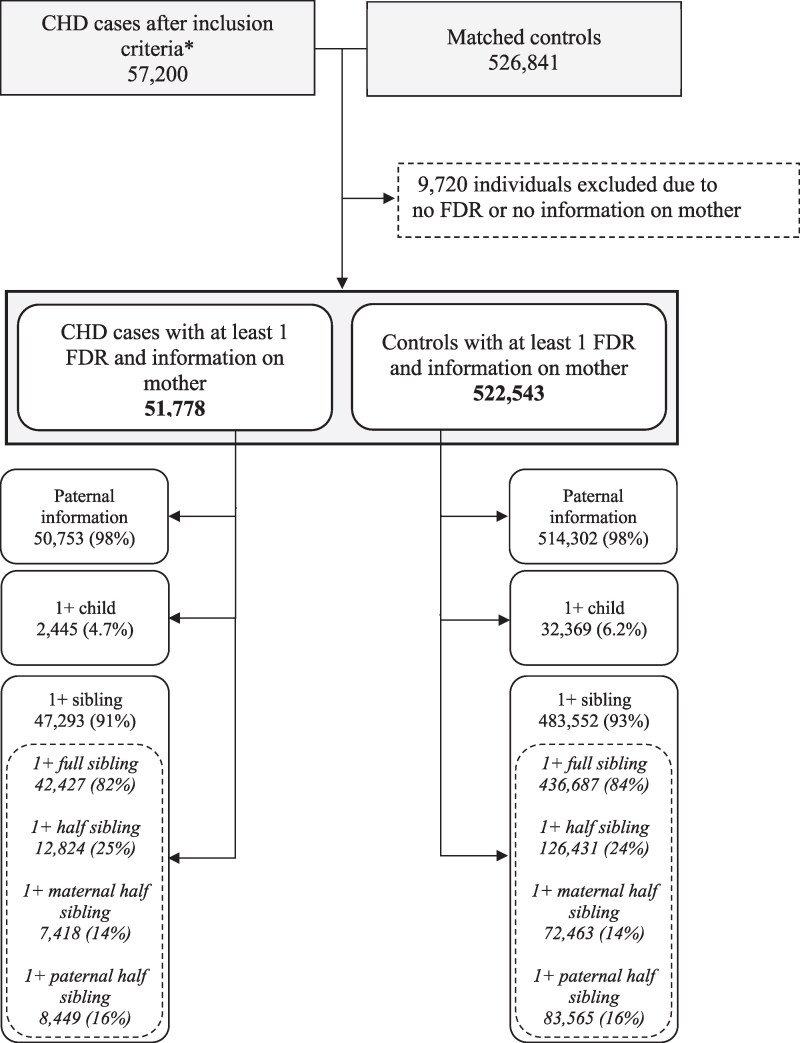
Flowchart of the final study population and family linkage by kinship type. This flowchart shows the number of cases—index individuals with congenital heart defects and their matched controls—retained for the final analysis after applying exclusion criteria (see also Supplementary data online, *Figure S1*). Matching was performed on birth year, sex, and county of residence at birth (10:1 ratio). Individuals (cases and controls) without identifiable first-degree relatives or maternal data in the Multi-Generation Register were excluded at the individual level. The lower section of the figure displays the proportion of cases and controls with available family linkage per kinship type, including paternal information, siblings, full and half-siblings (maternal and paternal), and offspring. Percentages were calculated within the group of congenital heart defect cases (*n* = 51 778) and controls (*n* = 522 543)

Subsequently, individuals with no identifiable FDR in the MGR or without a registered mother in the Swedish MBR were excluded. These exclusions were applied independently at the individual level, rather than at the matched set level, because adjustment for matching variables (birth year, sex) was performed in all models and the number of exclusions was small. The flowchart of the study population is presented in *Figure 1*.

### Exposure and outcome measures

The primary exposure was having at least one FDR with a diagnosis of CHD, defined as parents, full siblings, or offspring. Half-siblings, who share ∼25% of their genome, were not considered FDR but were analysed separately. Given their high prevalence in Swedish family structures, we also constructed a broader composite category, referred to as relatives, which included both FDR and half-siblings. Familial recurrence patterns were analysed by kinship type and by the number of affected relatives. The primary outcome was a diagnosis of CHD in the index individual. Congenital heart defect diagnoses were classified by severity according to EUROCAT (2018) criteria,^27^ with the addition of all CHD requiring surgical intervention before one year of age.

### Statistical analysis

Statistical analyses were conducted to evaluate associations between familial CHD and CHD in index individuals, to explore recurrence patterns across different kinships, and to assess dose–response relationships by number of affected relatives.

We used logistic regression to estimate odds ratios (ORs) and 95% confidence intervals (CIs) for the association between familial CHD and CHD in index cases. To account for intra-family correlation, robust standard errors were estimated using the sandwich estimator clustered on maternal personal identification number. Both conditional and unconditional logistic regressions were evaluated, yielding nearly identical results. Consistent with Mansournia *et al*.^28^ unconditional models adjusting for the matching variables were retained to allow inclusion of all matched sets and clustering of standard errors by maternal ID.

Covariates were pre-specified *a priori* using causal reasoning based on a directed acyclic graph informed by previous literature. The core adjustment set included sex, birth year, and county of residence at birth (matching variables), as well as family size (1–2, 3–5, or ≥6 relatives). Parental CHD and parental age at birth were added where relevant, such as in sibling exposure models, while in parental exposure models (e.g. paternal CHD–CHD in the index individual), CHD in the other parent was additionally included to account for correlated parental disease and avoid inflation of the estimated association.

The linearity of the only continuous covariate, birth year, was assessed using restricted cubic splines with 1–6 degrees of freedom, comparing model fit by Akaike’s and Bayesian information criteria. Both indicated that a linear specification provided the best fit. To confirm that categorizing family size did not bias results, the variable was also modelled as continuous and with splines (df = 3), yielding near-identical estimates.^29^

Separate models were fitted to examine patterns of familial recurrence. First, we assessed the association between the number of affected relatives (0, 1, 2, 3, or >3) and the odds of CHD in index individuals, using both categorical and continuous predictors, with the full cohort serving as a consistent reference group. We also repeated these analyses restricted to FDRs (parents, offspring, full siblings) to provide estimates based on the strict genetic definition. Second, we examined kinship-specific associations by fitting individual models for maternal, paternal, full sibling, half-sibling, and offspring CHD. Each of these models was restricted to individuals with at least one identifiable relative of the corresponding type and adjusted for parental age and, where applicable, CHD in the other parent. Finally, we evaluated dose–response relationships by number of affected relatives within specific kinships—full siblings, half-siblings, and offspring—modelled both categorically (0, 1, 2, ≥3) and continuously to assess linear trends. Where few individuals had more than three affected relatives, upper categories were collapsed (≥3) to improve estimate precision and to avoid reporting very small cell counts that could risk individual identification.

To assess whether maternal comorbidities modified the association between maternal CHD and CHD in the index individual, we fitted separate multivariable logistic regression models including an interaction term between maternal CHD and each comorbidity (diabetes, hypertension, and obesity). These comorbidities were selected *a priori* based on their well-established associations with CHD and consistent ascertainment in the national registers. All comorbidities were defined as pregestational, requiring at least two relevant ICD codes in the NPR ≥ 1 year before delivery or at the first antenatal visit in the MBR (see Supplementary data online, *Table S5*). Models were re-parameterized to obtain stratum-specific adjusted ORs (aORs) for maternal CHD within each comorbidity category. Interaction was evaluated on both multiplicative (ratio of ORs, ROR) and additive (relative excess risk due to interaction, RERI) scales with 95% CIs.^30^ All models were adjusted for sex, birth year, county of residence at birth, family size, and maternal age and used robust standard errors clustered by maternal identifier.

Sensitivity analyses included (i) reintroduction of individuals excluded due to register data reliability concerns; (ii) restriction to non-syndromic index cases; (iii) assessment of interaction between familial CHD and birth period (before vs after 2001); (iv) restriction to singleton births; and (v) restriction to severe CHD cases. Details are provided in Supplementary data online, *Appendix F*.

Analyses were conducted using Stata version 17 (StataCorp, College Station, TX, USA), with a two-sided significance level of *P* < .05.

## Results

A total of 51 778 index individuals with CHD (cases) and 522 543 matched population controls without CHD were included in the study (*Figure 1*). The prevalence of CHD by birth year is illustrated in Supplementary data online, *Figure S2*. Baseline characteristics and family structure are presented in *Table 1*, and the study population flowchart is shown in *Figure 1*. Cases and controls were similar in distribution of sex, year of birth, and parental age at childbirth. Notably, this balance was maintained despite exclusions being applied at the individual level rather than by matched sets.

**Table 1:** Population characteristics

		Cases (*n* = 51 778)	Controls (*n* = 522 543)
Sex, *n* (col %)	Male	25 841 (49.9)	260 615 (49.9)
	Female	25 937 (50.1)	261 928 (50.1)
Birth year, *n* (col %)	1987–91	4965 (9.6)	50 189 (9.6)
	1992–96	5634 (10.9)	57 151 (10.9)
	1997–2001	6133 (11.8)	62 188 (11.9)
	2002–06	10 235 (19.8)	103 282 (19.8)
	2007–11	11 111 (21.5)	112 340 (21.5)
	2012–17	13 700 (26.5)	137 393 (26.3)
Severe CHD, *n* (col %)	Yes	7229 (14.0)	
	No	44 549 (86.0)	
Repaired CHD, *n* (col %)	Yes	12 636 (24.4)	
	No	39 142 (75.6)	
Any genetic abnormality or ECA, *n* (col %)	Yes	12 152 (23.5)	
	No	39 626 (76.5)	
Family size, *n* (col %)	Small	4417 (8.5)	38 178 (7.3)
	Medium	40 662 (78.5)	421 289 (80.6)
	Large	6699 (13.0)	63 076 (12.1)
Maternal birth year, mean (SD)		1974 (9)	1974 (9)
Maternal age at childbirth, *n* (col %)	<25	7360 (14.2)	72 333 (13.8)
	25–34	32 419 (62.6)	336 873 (64.5)
	35–39	9336 (18.0)	91 195 (17.5)
	≥40	2663 (5.1)	22 142 (4.2)
	Mean (SD)	30.3 (5.2)	30.3 (5.2)
Paternal birth year, mean (SD)		1971 (10)	1971 (10)
Paternal age at childbirth, *n* (col %)	<25	3157 (5.9)	30 554 (6.1)
	25–34	27 764 (54.8)	286 280 (53.6)
	35–39	11 836 (23.2)	121 440 (22.9)
	≥40	9021 (16.1)	84 269 (17.4)
	Mean (SD)	33.3 (6.3)	33.3 (6.3)

This table summarizes demographic and familial characteristics of individuals diagnosed with congenital heart defects and their matched controls. Distributions of sex, birth year, and family size [defined as the total number of identifiable relatives and categorized as small (1–2 relatives), medium (3–5), or large (≥6)], as well as parental birth year and age at childbirth, are presented. Percentages are column-wise. Severe and repaired CHD are only applicable to the CHD group. Proportions of genetic or extracardiac abnormalities refer to the presence of any recorded genetic syndrome or major extracardiac malformation. ‘Cases’ refer to index individuals diagnosed with CHD; ‘controls’ refer to matched individuals without CHD. A 1:10 matching ratio was used for cohort construction; minor deviations in the final numbers reflect individual-level exclusions of participants without identifiable first-degree relatives or missing maternal identifiers.

CHD, congenital heart defect; FDR, first-degree relative; SD, standard deviation; ECA, extracardiac abnormality.

Among CHD cases, 13.7% were classified as severe disease, 24.4% had undergone surgical repair, and 23.5% were diagnosed with either genetic or extracardiac abnormality (*Table 1*; Supplementary data online, *Appendix E* and *Table S6*). Family linkage was nearly complete, with over 98% of participants having identifiable parents and more than 91% having at least one sibling; family structures (e.g. number and types of relatives) were similar between cases and controls.

### Familial burden of congenital heart defects by number of affected relatives

**Figure 2:**
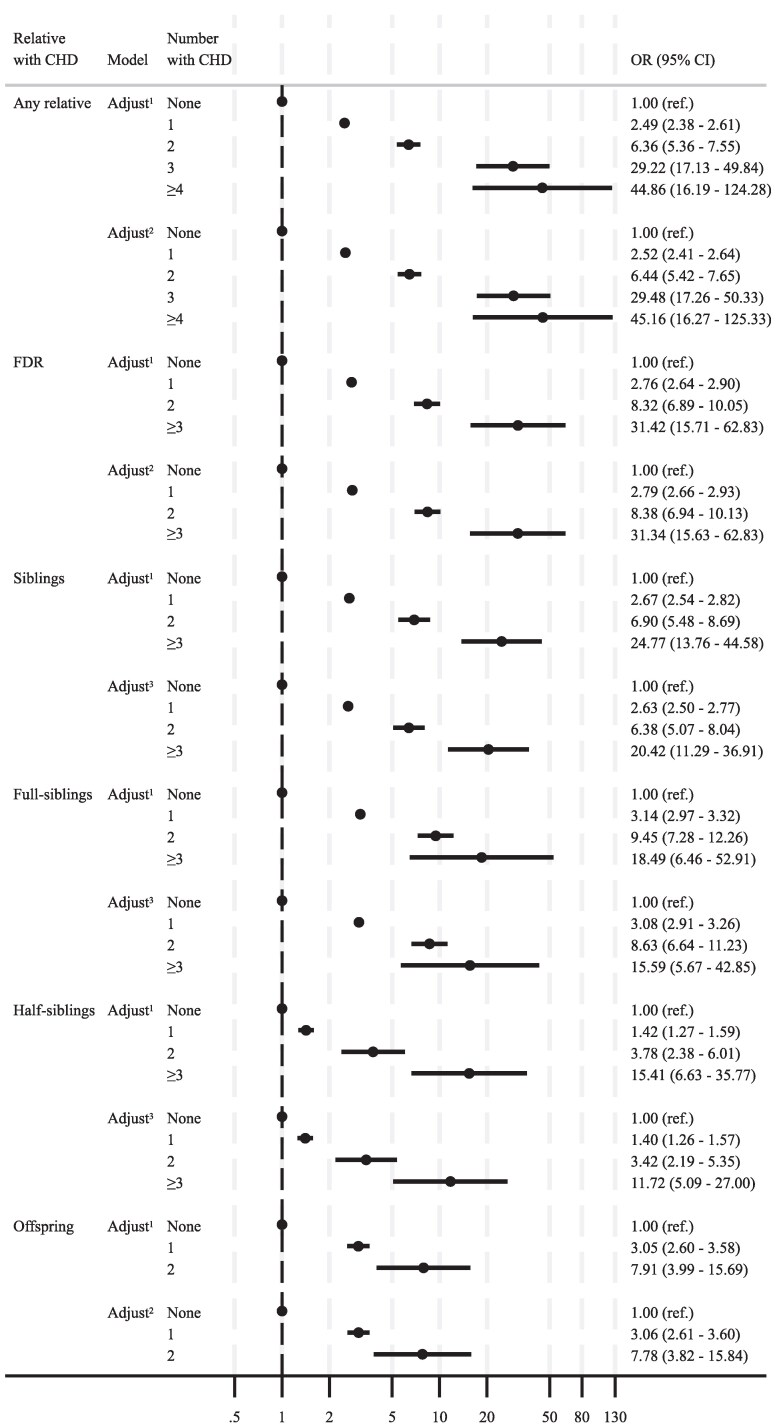
Dose–response relationship between the number of affected relatives and odds of congenital heart defect. Forest plot showing adjusted odds ratios and 95% confidence intervals for congenital heart defects according to the number of affected relatives by kinship group: any relative (including first-degree relative and half-siblings), any first-degree relative, any sibling, full sibling, half-sibling, and offspring. Models labelled ‘Adjust¹’ include sex, birth year, and county of residence at birth; ‘Adjust²’ additionally includes family size (1–2, 3–5, ≥6 relatives); and ‘Adjust³’ also adjusts for parental age and parental CHD, applicable to sibling models only. Estimates were obtained from logistic regression models with robust standard errors clustered by maternal identifier

A higher proportion of cases had one or more affected relatives compared with controls (8.9% vs 3.5%; *Table 2*). The presence of at least one affected relative was associated with significantly higher odds of CHD (aOR 2.71, 95% CI 2.60–2.83) (*Figure 2*). However, most cases (91%) had no affected relatives, indicating that the majority of CHD is sporadic (*Table 2*).

**Table 2:** Distribution of index individuals by number of affected relatives with congenital heart defect

Number of relatives with CHD	Cases	Controls
Any type (FDR + half-siblings)		
1	4135/51 778 (7.99)	17 763/522 543 (3.40)
2	387/51 778 (0.75)	652/522 543 (0.12)
3	79/51 778 (0.15)	29/522 543 (0.00)
4 or more	25/51 778 (0.05)	6/522 543 (0.00)
FDR		
1	3746/51 778 (7.23)	14 462/522 543 (2.77)
2	349/51 778 (0.67)	448/522 543 (0.09)
3 or more	58/51 778 (0.11)	18/522 543 (0.00)
Siblings		
0	43 581/47 293 (92.15)	469 386/483 552 (97.07)
1	3405/47 293 (7.20)	13 747/483 552 (2.84)
2	252/47 293 (0.53)	395/483 552 (0.08)
3 or more	55/47 293 (0.12)	24/483 552 (0.00)
Full siblings		
0	39 221/42 427 (92.44)	426 111/436 687 (97.58)
1	2972/42 427 (7.00)	10 315/436 687 (2.36)
2	217/42 427 (0.51)	251/436 687 (0.06)
3 or more	17/42 427 (0.04)	10/436 687 (0.00)
Half-siblings		
0	12 269/12 824 (95.67)	122 780/126 431 (97.11)
1	505/12 824 (3.94)	3547/126 431 (2.81)
2	36/12 824 (0.28)	95/126 431 (0.08)
3 or more	14/12 824 (0.11)	9/126 431 (0.01)
Offspring		
0	2237/2445 (91.49)	31 443/32 369 (97.14)
1	195/2445 (7.98)	903/32 369 (2.79)
2	13/2445 (0.53)	23/32 369 (0.07)

This descriptive table presents the number and percentage of CHD cases and matched controls with 1, 2, 3, or ≥4 affected relatives, stratified by relationship type: any type, FDR (first-degree relatives), siblings, full siblings, half-siblings, and offspring. Denominators (N) include only those with identifiable relatives of the corresponding kinship type.

CHD, congenital heart defect; FDR, first-degree relative.

A clear dose–response pattern was observed. Compared with those with no affected relatives, the adjusted odds of CHD were 2.52 (95% CI 2.41–2.64) with one affected relative, 6.44 (95% CI 5.42–7.65) with two, 29.48 (95% CI 17.26–50.33) with three, and 45.16 (95% CI 16.27–125.33) with four or more. When modelled continuously, each additional affected relative was associated with a 2.55-fold increase in odds of CHD (95% CI 2.46–2.64; *P* < .001).

Very similar results were obtained when restricting to strict FDR (parents, offspring, full siblings): 2.76 (95% CI 2.64–2.90) with one, 8.32 (95% CI 6.89–10.05) with two, and 31.42 (95% CI 15.71–62.83) with three or more.

### Kinship-specific recurrence patterns

When stratifying by kinship, CHD recurrence patterns mirrored the degree of genetic relatedness. The adjusted odds were similarly high for individuals with an affected full sibling (aOR 3.22; 95% CI 3.05–3.40), mother (aOR 3.12; 95% CI 2.81–3.47), or offspring (aOR 3.18; 95% CI 2.72–3.72). In contrast, lower odds were observed for individuals with an affected father (aOR 2.25; 95% CI 2.01–2.52), maternal half-sibling (aOR 1.67; 95% CI 1.40–1.98), or paternal half-sibling (aOR 1.32; 95% CI 1.16–1.51) (*Table 3*).

**Table 3:** Association between number and type of affected relatives and odds of congenital heart defect in index individuals

	Cases	Controls	Adjusted OR (95% CI)	Further adjusted OR (95% CI)
Relatives with CHD				
0 relative, *n* (col %)	47 152/51 778 (91.07)	504 093/522 543 (96.47)	1.00 (reference)	
≥1 relatives, *n* (col %)	4626 (8.93)	18 450 (3.53)	2.68 (2.57–2.80)	2.71 (2.60–2.83)
≥1 FDRs, *n* (col %)	4153 (8.02)	14 928 (2.86)	2.97 (2.83–3.10)	2.99 (2.86–3.14)
By kinship				
Mother	539/51 778 (1.04)	1756/522 543 (0.34)	3.12 (2.80–3.46)	3.12 (2.81–3.47)
Father	413/50 753 (0.81)	1860/514 302 (0.36)	2.26 (2.02–2.53)	2.25 (2.01–2.52)
Full sibling	3206/42 427 (7.56)	10 576/436 687 (2.42)	3.30 (3.12–3.48)	3.22 (3.05–3.40)
Maternal half-sibling	292/7418 (3.94)	1693/72 463 (2.34)	1.71 (1.43–2.03)	1.67 (1.40–1.98)
Paternal half-sibling	267/8449 (3.16)	1987/83 565 (2.38)	1.33 (1.17–1.53)	1.32 (1.16–1.51)
Offspring	208/2445 (8.51)	926/32 369 (2.86)	3.18 (2.70–3.69)	3.18 (2.72–3.72)

Odds ratios and 95% confidence intervals for congenital heart defect in cases, based on the presence of any affected relative and by kinship type of the affected relative. Logistic regression models were adjusted for sex, birth year, and county of residence at birth. Further adjusted models additionally included family size [categorized as small (1–2 relatives), medium (3–5 relatives), or large (≥6 relatives)] and kinship-specific covariates as follows: maternal age and paternal CHD for mother; paternal age and maternal CHD for father; maternal age, paternal age, and parental CHD for full and half-siblings; and family size only for offspring. Analyses were restricted to individuals with at least one identifiable relative of the corresponding kinship type to avoid misclassification due to missing family data. Robust standard errors clustered by maternal identifier were used. Relatives includes FDR and half-siblings. Reference category is individuals with no affected relatives (OR = 1.00).

CHD, congenital heart defect; FDR, first-degree relative; OR, odds ratio; CI, confidence interval.

### Dose–response by number of affected siblings

The dose–response pattern persisted within sibling subgroups (*Figure 2*). For full siblings, aORs increased from 3.08 (95% CI 2.91–3.26) for one affected sibling to 8.63 (95% CI 6.64–11.23) for two and 15.59 (95% CI 5.67–42.85) for three or more. The corresponding aORs for half-siblings were lower in magnitude but followed a similar pattern: 1.40 (95% CI 1.26–1.57), 3.42 (95% CI 2.19–5.35), and 11.72 (95% CI 5.09–27.0), respectively (*Figure 2*).

When modelled continuously, each additional affected sibling was associated with a nearly three-fold increase in odds for full siblings (aOR 3.03; 95% CI 2.89–3.18) and 1.5-fold for half-siblings (aOR 1.53; 95% CI 1.38–1.69). A similar trend was observed when full and half-siblings were combined (aOR 2.61; 95% CI 2.49–2.72). All trends were statistically significant (*P* < .001).

### Dose–response by number of affected offspring

Among the relatively small subset of index cases with offspring, having one or more children with CHD was associated with a higher likelihood of CHD in the parent. Individuals with one affected child had more than three-fold higher odds of having CHD themselves (aOR 3.06; 95% CI 2.61–3.60), while those with two affected children had nearly eight-fold higher odds (aOR 7.78; 95% CI 3.82–15.84), compared with those with no affected children (*Figure 2*). The odds of CHD increased progressively with the number of affected offspring (*P* for trend < .001), with an estimated OR of 3.16 (95% CI 2.70–3.70) per affected child.

This analysis was used to explore familial aggregation in the opposite generational direction, from index cases to their offspring. Since offspring CHD necessarily occurred after the index case’s birth, the association does not imply causality but rather reflects underlying shared genetic or familial predisposition.

### Maternal comorbidities and interaction with maternal congenital heart defects

Because maternal diabetes, obesity, and hypertension are established risk factors for CHD in the offspring, we assessed whether these comorbidities modified the association between maternal CHD and CHD in the index case (*Table 4*). Among mothers without diabetes, maternal CHD was associated with more than a three-fold increase in the odds of CHD in the index case (aOR 3.15; 95% CI 2.83–3.51). Among diabetic mothers, this association was attenuated (aOR 1.14; 95% CI 0.42–3.08). On the multiplicative scale, the ROR was 0.37 (95% CI 0.14–0.99; *P* = .048), indicating a significantly weaker effect of maternal CHD among diabetic mothers. The relative excess risk due to interaction (RERI = −1.71; 95% CI −4.89–1.47; *P* = .291) showed no evidence of additive interaction.

**Table 4:** Interaction between maternal congenital heart defect and maternal comorbidities in relation to congenital heart defect in index individuals

Maternal comorbidity	CHD in mother	CHD in index individual^a^, yes/no	Adjusted OR (95% CI) for CHD in mother within strata of comorbidity
Diabetes			
No	No	50 615/518 486	1.00 (ref)
	Yes	534/1 740	3.15 (2.83–3.51)
Yes	No	624/2 301	1.00 (ref)
	Yes	5/16	1.14 (0.42–3.08)
Multiplicative scale: ratio of ORs = 0.37 (0.14–0.99); *P* = .048 Additive scale: RERI = −1.71 (−4.89–1.47); *P* = .291			
Hypertension			
No	No	51 122/520 180	1.00 (ref)
	Yes	534/1 742	3.13 (2.81–3.47)
Yes	No	117/607	1.00 (ref)
	Yes	5/14	1.94 (0.57–6.53)
Multiplicative scale: ratio of ORs = 0.62 (0.18–2.10); *P* = .442 Additive scale: RERI = −0.33 (−4.81–4.15); *P* = .885			
Obesity			
No	No	49 554/507 719	1.00 (ref)
	Yes	518/1 707	3.12 (2.80–3.48)
Yes	No	1 685/13 068	1.00 (ref)
	Yes	21/49	3.30 (1.94–5.61)
Multiplicative scale: ratio of ORs = 1.06 (0.62–1.82); *P* = .841 Additive scale: RERI = 0.90 (−1.41–3.21); *P* = .448			

This table presents adjusted odds ratios (aORs), 95% confidence intervals (CIs), and interaction statistics from three separate logistic regression models assessing whether maternal comorbidities (diabetes, hypertension, obesity) modify the association between maternal CHD and CHD in the index individual. For each comorbidity, models include an interaction term between maternal CHD and the comorbidity. Stratum-specific aORs for maternal CHD are reported within comorbidity categories (yes/no). Interaction on the multiplicative scale is summarized by the ratio of odds ratios (ROR) with *P*-values and on the additive scale by the relative excess risk due to interaction (RERI) with 95% CIs. All models were adjusted for sex, birth year, family size, maternal age, and county of residence at birth and used robust standard errors clustered by maternal identifier.

CHD, congenital heart defect; aOR, adjusted odds ratio; CI, confidence interval.

^a^‘CHD in index, yes’ corresponds to cases and ‘CHD in index, no’ to matched controls.

For obesity, the association remained consistent across strata: aOR 3.12 (95% CI 2.80–3.48) among non-obese and 3.30 (95% CI 1.94–5.61) among obese mothers. The ROR was 1.06 (95% CI 0.62–1.82; *P* = .841) and the RERI 0.90 (95% CI −1.41–3.21; *P* = .448), indicating no interaction on either scale.

For hypertension, maternal CHD was associated with aOR 3.13 (95% CI 2.81–3.47) among normotensive and 1.94 (95% CI 0.57–6.53) among hypertensive mothers, although the wide CIs reflect limited precision due to small numbers. The ROR was 0.62 (95% CI 0.18–2.10; *P* = .442) and the RERI −0.33 (95% CI −4.81–4.15; *P* = .885), providing no evidence of effect modification.

Together, these results suggest a potential modification of the association between maternal CHD and CHD in index cases by maternal diabetes, whereas no such effect was observed for obesity or hypertension. These findings support the role of maternal CHD as an independent contributor to the risk of CHD in index cases.

### Sensitivity analyses

All sensitivity analyses are presented in Supplementary data online, *Tables S7*–*S10* and *Figure S3*. Results remained consistent after reintroducing individuals initially excluded due to potential diagnostic misclassification (e.g. isolated, implausible, or likely maternal records), included to assess the impact of our exclusion criteria (see Supplementary data online, *Figure S3*). Findings were also unchanged when restricting to non-syndromic CHD cases (i.e. without extracardiac anomalies or genetic syndromes) (see Supplementary data online, *Table S7*), analysing only individuals with severe CHD (see Supplementary data online, *Table S9*), and limiting to singletons (see Supplementary data online, *Table S10*).

To assess temporal effects, we tested for interaction by birth period (before vs after 2001, when outpatient diagnoses were added to the NPR). The association between CHD in any relative and in the index individual was slightly higher after 2001 (aOR 2.80 vs 2.49), with evidence of positive additive interaction (RERI = 1.12, *P* = .011) but a smaller proportional effect on the multiplicative scale (ROR = 0.29, 95% CI 0.06–0.51; *P* = .013). A similar pattern was observed for maternal half-siblings (RERI = 0.55, *P* = .018; ROR = 1.47, *P* = .020), whereas no significant interactions were detected for other kinship types (see Supplementary data online, *Table S8*).

## Discussion

This nationwide, population-based study confirms familial clustering of CHD, with a clear dose–response relationship and kinship-specific gradients supporting a heritable component (*Structured Graphical Abstract*). Our findings align with previous evidence that CHD aggregates in families, particularly among close genetic relatives.^16,17,19,31,32^

We observed several consistent patterns across analyses. First, the odds of CHD increased progressively with the number of affected FDRs, suggesting a dose–response pattern of familial susceptibility likely driven by shared genetic factors. This is supported by genomic studies that have identified inherited variants contributing to CHD risk, together with sporadic *de novo* variants that primarily explain isolated cases rather than familial clustering.^9,33–35^

Second, the association varied by type of kinship. The strongest associations were observed for full siblings, mothers, and offspring—relatives who share approximately 50% of their genetic material with the index individual. This kinship-specific gradient further supports a genetic contribution and mirrors recurrence patterns described by Ellesøe *et al*.,^36^ who reported high variability in concordance and discordance across families.

Third, a dose–response pattern was evident when comparing full siblings with half-siblings, with stronger associations in the former group, further reinforcing the role of genetic relatedness. This is in line with previous findings by Brodwall *et al*.^18^ who also reported stronger associations among full siblings, supporting the role of shared genetics in CHD recurrence.

Similar trends were observed when evaluating the number of affected offspring. This suggests familial clustering independent of generational direction. Although cross-generational associations should be interpreted with caution due to potential confounding by shared environment and reproductive fitness, the consistency across generations reinforces the heritable component of CHD.

A particularly notable finding was the stronger association observed through the maternal line, compared with the paternal line. This maternal–paternal asymmetry—also reported in prior studies^37–40^—may reflect maternal-specific mechanisms, such as intrauterine influences, mitochondrial inheritance, sex-specific epigenetic regulation, or maternal-effect genes involved in early cardiac development.^14,41–45^ The higher odds of CHD among those with maternal half-siblings, compared with paternal half-siblings—despite equal genetic sharing—aligns with the hypothesis that maternal-specific factors contribute to CHD risk.

In our data, the aOR for maternal CHD was 3.12, compared with 2.25 for paternal CHD, and this difference was statistically significant (*P* < .0001). Øyen *et al*.^37^ using a prospective cohort design and estimating relative risks, reported a higher maternal than paternal recurrence risk (recurrence risk ratio 1.82). Despite differences in design and effect measures, both our findings and prior studies consistently support stronger maternal than paternal transmission of CHD.

Part of this asymmetry may reflect differences in how maternal and paternal data are recorded in health registers. Uncertainty in biological paternity could attenuate paternal associations. Maternal identity is reliably documented through birth records, whereas paternal identity is based on legal registration and may not always reflect biological paternity. However, a recent Swedish nationwide study estimated the misattributed paternity rate at approximately 1.7%, decreasing to around 1% in recent years.^46^ Additionally, differential detection or recording of CHD diagnoses in women may play a role, given their generally higher healthcare utilization. To reduce misclassification, we excluded CHD diagnoses deemed potentially spurious based on care level and clinical trajectory (see Supplementary data online, *Appendix B*). While this likely improved specificity, it may have disproportionately excluded milder maternal cases, potentially attenuating the observed maternal associations. However, our sensitivity analyses, which retained these excluded diagnoses, did not materially alter dose–response or kinship-specific estimates. Finally, Øyen *et al*. found that adjusting for parity did not influence the maternal–paternal difference, suggesting that differential fertility is unlikely to account for this pattern.

To further explore maternal influences, we examined whether the association between maternal CHD and CHD in index cases was modified by maternal diabetes, obesity, or hypertension. Both multiplicative and additive scales were examined to capture different dimensions of interaction. While multiplicative interaction assesses whether the relative effect of maternal CHD differs across comorbidity strata, additive interaction (quantified as the RERI) is more informative for causal interpretation and public health impact.^30^ In this study, the absence of significant additive interaction suggests that co-occurrence of maternal CHD and maternal comorbidities does not produce a synergistic increase in offspring CHD risk beyond the sum of their individual effects. The association appeared attenuated among mothers with diabetes but remained consistent among those with and without obesity. For hypertension, the estimates were lower among hypertensive mothers, but the wide CIs and lack of statistical significance suggest limited power to detect interactions in this subgroup.

The attenuation observed in diabetic mothers may reflect overlapping mechanisms. One possibility is risk saturation, where baseline CHD risk is already elevated due to maternal CHD, limiting any additional contribution from diabetes. Alternatively, both maternal CHD and diabetes may influence similar developmental pathways, reducing any observed interaction.^47^ Additionally, differences in prenatal surveillance of diabetic mothers may result in more uniform CHD detection, irrespective of maternal CHD status. This attenuation does not imply a protective effect, but a weaker relative contribution of maternal CHD in this subgroup. The consistently elevated odds among non-diabetic mothers support the interpretation that maternal CHD is an independent contributor to CHD risk in offspring. While these interpretations align with proposed biological mechanisms, they remain hypotheses warranting confirmation in molecular or prospective studies.

We explored temporal trends to account for evolving register coverage and the increasing detection of predominantly mild CHD. The association between familial CHD and CHD in index individuals was modestly yet statistically significantly higher among those born after 2001, coinciding with the inclusion of outpatient diagnoses in the Swedish NPR. This finding likely reflects improved ascertainment of milder, non-hospitalized cases in more recent cohorts. On the additive scale, the positive interaction suggests that being born after 2001 and having an affected relative jointly increased the number of detected CHD cases beyond what would be expected from their individual effects. In contrast, the smaller proportional difference on the multiplicative scale indicates that the relative strength of the familial association was somewhat attenuated in later years—consistent with a dilution effect from better detection of sporadic and milder defects. Interaction was statistically significant only for maternal half-siblings, possibly reflecting differences in prenatal detection, care-seeking patterns, or exposure to shared maternal factors. For other kinship types, the familial associations remained stable across birth cohorts, supporting that the observed clustering largely reflects true familial risk rather than artefacts of changing register sensitivity.

### Strengths and limitations

Strengths of this study include the use of a large, well-characterized national cohort with near-complete coverage of the Swedish population and validated family linkages across three decades. This scale enabled precise estimation of familial recurrence in a contemporary setting and provided power to quantify dose–response relationships and kinship-specific associations, including separate analyses of maternal and paternal half-siblings, not previously reported. The long study period also spanned substantial changes in CHD epidemiology, driven by improved register coverage and diagnostic practices. Despite these shifts, recurrence patterns remained consistent over time, strengthening the generalizability of our findings. Importantly, this is the first population-based study sufficiently powered to assess whether maternal comorbidities modify recurrence patterns. Moreover, our carefully applied exclusion criteria aimed to reduce misclassification and improve diagnostic validity compared with earlier register-based studies.^48^ Multiple sensitivity analyses also confirmed that the findings were robust across subgroups, severity strata, and calendar periods. While broadly consistent with previous research, our study adds novel detail by providing dose–response estimates across diverse family structures in a contemporary population-based setting. Lesion-specific recurrence patterns, which are of particular clinical and scientific importance, will be reported separately in forthcoming work based on the same study population.

Several limitations should also be noted. First, CHD diagnoses derived from national health registers may be subject to misclassification, although the risk was reduced by applying a hierarchical classification strategy of exclusion criteria based on diagnostic specificity and clinical plausibility. Second, genetic data were not available, limiting our ability to distinguish between inherited genetic variants and non-genetic familial factors. Third, although all index individuals were born in Sweden, differences in ancestry and parental country of birth could influence recurrence patterns through variation in genetic background, diagnostic pathways, and completeness of recording of parental CHD. As genetic ancestry is not captured in Swedish registers, country of birth is only a crude proxy and cannot adequately reflect genetic or social factors such as consanguinity. Any differences in parental country of birth are likely to be distributed equally across cases and controls, minimizing systematic bias in relative comparisons. Nevertheless, ancestry remains an important area for future research requiring study designs with detailed genetic and sociodemographic data. Fourth, data on pregnancy terminations following prenatal CHD diagnoses were not available. This likely results in underestimation of familial risk, especially for maternal transmission, where increased surveillance may lead to earlier detection and selective termination. Although sensitivity analyses across birth years and CHD severity showed stable results, this remains a limitation that may affect generalizability to all conceptions. In addition, register-based data on maternal comorbidities may also be incomplete, particularly for conditions managed primarily in outpatient care. Although we supplemented these data with information from the MBR to improve ascertainment, underreporting may persist and could reduce power to detect interactions. This is particularly relevant for hypertension, where few cases were observed in the maternal CHD stratum. Similarly, very rare exposure categories (e.g. individuals with ≥3 affected relatives) may have yielded inflated or imprecise estimates due to sparse-data bias. Another limitation is that our study design did not allow modelling of birth-order effects. Unlike cohort studies that define recurrence risk based on older-to-younger sibling order, our approach focused on overall familial clustering irrespective of temporal sequence and should therefore not be interpreted as prospective recurrence risk. This restricts causal interpretation across generations or siblings but enhances generalizability by capturing a wider range of family configurations and remains clinically relevant for understanding recurrence patterns regardless of directionality. Furthermore, as a case-control study, our design cannot provide absolute recurrence risks. Our findings complement cohort studies by describing clustering in a nationwide population. Finally, residual confounding from unmeasured familial or environmental factors cannot be excluded.

## Conclusion

In summary, this nationwide study confirms that CHD clusters within families. The likelihood increases according to both the number and genetic closeness of affected relatives. Recurrence was stronger through the maternal line, supporting the contributions of both inherited genetic factors and maternal-specific influences. Despite substantial changes in register coverage and CHD prevalence over time, familial associations remained stable, underscoring the robustness of the observed clustering. These findings highlight the clinical importance of detailed family history in risk assessment and the need to integrate genomic data in future research to better elucidate recurrence mechanisms and improve counselling. Linkage with genetic and foetal diagnostic data may further clarify underlying pathways and refine estimation of recurrence risk.

## Supplementary Material

ehaf1048_Supplementary_Data

## Declarations

### Data Availability

The data underlying this article cannot be shared publicly due to ethical reasons.

### Pre-registered Clinical Trial Number

None supplied.
