# Supplementary material for: Congenital heart defects: familial recurrence patterns in Sweden
Source: Eur Heart J. 2026 Jan 8;47(29):3905–16. doi: 10.1093/eurheartj/ehaf1048 (PMC13429267; doi:10.1093/eurheartj/ehaf1048)
Supplement: ehaf1048_Supplementary_Data [file ehaf1048_supplementary_data.pdf]

Supplementary Material

Table of contents

Supplementary Material 1

Appendix A: Description of Swedish National Registers Used for Data Collection 2

Appendix B: Exclusion Criteria to Minimize Misclassification Bias 3

Appendix C: Diagnostic Code Tables 8

Appendix D: CHD Prevalence by Birth Year 11

Appendix E: Frequency of Genetic Syndromes and Extracardiac Anomalies 12

Appendix F: Sensitivity Analyses 13

Inclusion of Cases Excluded for Registry Data Reliability concerns 13

Exclusion of index individuals with documented genetic syndromes or extracardiac anomalies 15

Interaction Between Familial CHD and Birth Period 16

Analysis restricted to severe CHD 18

Analysis restricted to singleton births 19

Appendix A: Description of Swedish National Registers Used for Data Collection

Data were retrieved from multiple Swedish national registers:

I. ***The National Patient Register (NPR)***, established in 1964, with nationwide coverage since 1987. It captures primary and secondary discharge diagnoses and surgical procedures for hospital admissions. Since 2001, it includes approximately 80% of all non-primary outpatient visits. Missing visits are primarily from private practice. Additionally, all hospitalizations, surgeries, and catheter interventions at Sweden's cardiothoracic surgery clinics have been recorded since 1970.

II. ***The Multi-Generation Register (MGR)***, available for individuals born since 1932 and registered in Sweden since 1961, allowed linkage of index individuals to parents, siblings, and offspring.

III. ***The Swedish Medical Birth Register (MBR)***, established in 1973, includes data on pregnancies, deliveries, and newborns. It covers live births and stillborns with gestational age ≥22+0 weeks (≥28+0 before July 1, 2008).

IV. ***The Cause of Death Register*** has recorded all deaths in Sweden since 1952. These datasets were linked using the unique personal identification number assigned to each Swedish resident.

Appendix B: Exclusion Criteria to Minimize Misclassification Bias

To prioritize diagnostic accuracy, we implemented a hierarchical classification system based on the level of care where the diagnosis was made:

- Level 1: Pediatric surgical centers during initial surgery
- Level 2: Pediatric heart centers/university hospitals
- Level 3: County hospital pediatric clinics
- Level 4: General pediatric outpatient units and small county hospitals

To enhance diagnostic accuracy and minimize misclassification bias inherent in register-based studies, we applied a set of predefined exclusion criteria developed through systematic review of registry data, aimed at identifying patterns suggestive of misclassification.

1) **Physiological/Transitional Diagnoses and Specific Exclusions**

   a) **Preterm Infants with Patent Ductus Arteriosus (PDA)**

      Isolated PDA diagnosis co-occurring with prematurity codes.

      Rationale: PDA in preterm is not considered being true CHD.

      **All VSDs were classified as CHD even in preterms, as registry data do not allow reliable differentiation of small muscular defects that may close spontaneously.*

   b) **Early-Life Physiological Findings Without Follow-Up**

      Isolated diagnoses of patent foramen ovale (PFO), pulmonary stenosis (PS), pulmonary branch stenosis, PDA (non-preterm) before 12 weeks of age, without any follow-up beyond 12 weeks of age and without surgery or catheter intervention.

Rationale: These findings likely represent transient physiological conditions that do not warrant a CHD classification. This approach also addresses the coding overlap of PFO and secundum ASD under ICD-9 and minimizes misclassification of physiological PFOs as ASDs.

c) **Isolated Unspecified Malformations of the Circulatory System**

Isolated diagnosis of unspecified malformations of the circulatory system without surgery or catheter-based intervention

Rationale: These codes are too non-specific making them unreliable for case classification.

d) **Isolated Atrioventricular Block (AVB)**

Rationale: This disease has other underlying mechanism and is not considered being a structural CHD

2) **Fetal CHD Misattributed to Mothers**

Excluded if:

i) Female aged ≥15 years

ii) CHD diagnosis appears only within 30 weeks prior to delivery (date identified in MBR) or diagnosis for miscarriage, termination, or IUFD beyond 12 gestational weeks

iii) Exceptions made for specific CHD codes that are unlikely to reflect fetal misclassification

Rationale: These cases likely reflect a fetal diagnosis erroneously recorded under the mother's ID.

3) **Registry Data Reliability**

a) **Single record of moderate or severe CHD without follow-up**

Single record of moderate or severe CDH from any hospital level without follow-up and alive >1 year of age. *Rationale: A single unconfirmed diagnosis of moderate or severe CHD is likely to reflect miscoding.*

b) **Records only at level 4 hospitals**

Excluded if:

i) There is a single record only at level 4 hospital with no additional follow up and the patient is alive more than 2 years after the diagnosis.

Rationale: Although the patient may have CHD, this pattern suggests a likely coding error or misclassification due to absence of expected referral or follow-up.

ii) Multiple entries (≥2 visits) of moderate/severe CHD without collaboration with higher levels of care and alive > 2 years of age

Rationale: The patient may have CHD, but the specific severe diagnosis is likely incorrect. Severe CHD would typically trigger referral to a specialist center, which is absent in these cases.

c) **Fatal CHD without intervention**

Diagnosis of lethal CHD but survived > 2 years without any surgery or catheter-based intervention recorded. *Rationale: Survival without treatment is inconsistent with these diagnoses, suggesting misclassification.*

<u>Note (applies to 3a and 4c):</u>

If a patient had only one visit with a lethal or severe CHD code meeting the exclusion criteria, the entire case was excluded.

If a patient had multiple total visits, but only one of those visits included a lethal or severe CHD code, the patient was retained, and only that specific visit (row) was excluded.

These exclusions were consistently applied to index individuals and their FDRs. Relatives meeting criteria remained in the dataset but were not considered to have CHD in the analysis. See Table S1 for diagnostic codes and Supplementary Figure S1 for the flowchart of exclusions.

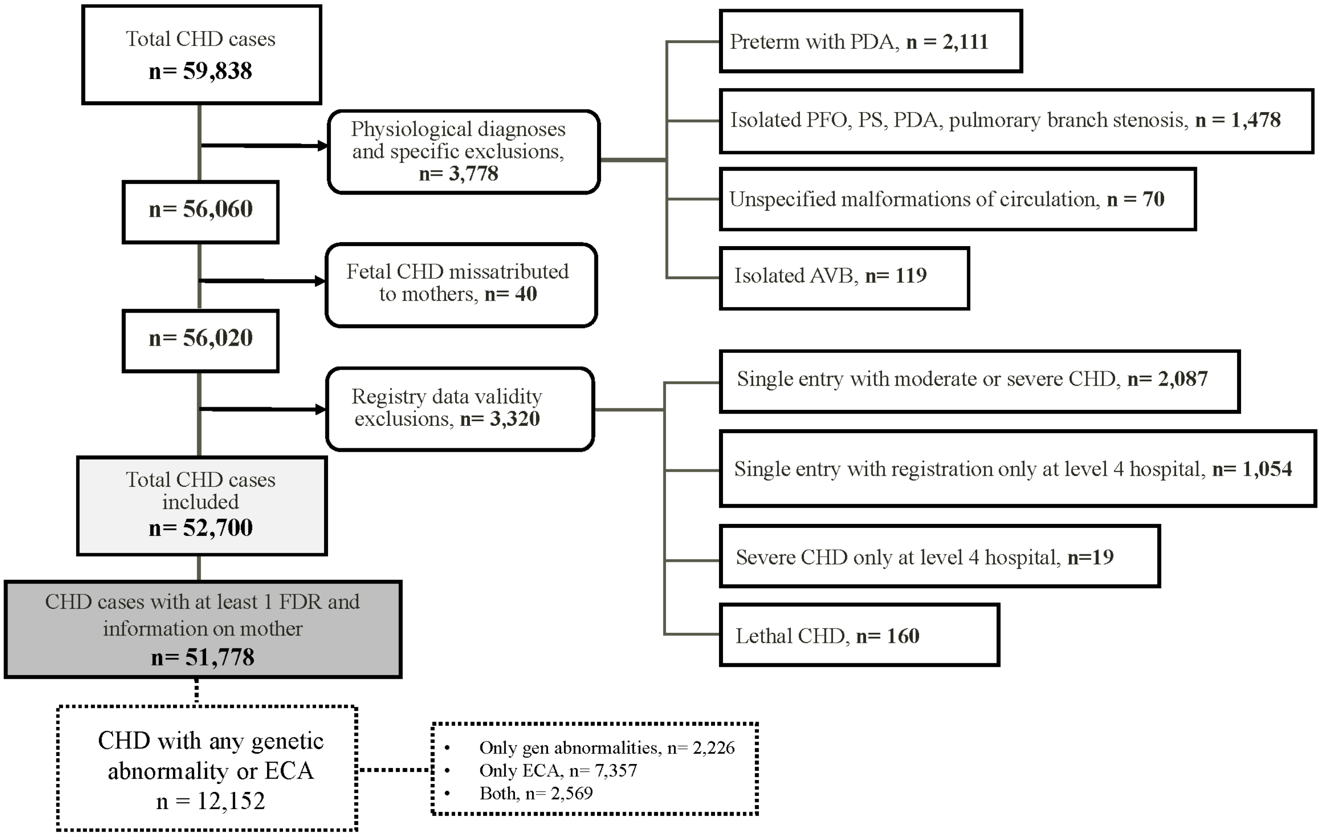

Figure S1. Flowchart of population assembly. Depicts stepwise application of exclusion criteria for index cases as described in Appendix B. For each excluded CHD case, the entire matched stratum, including all 10 matched controls, was excluded. The resulting cohort of 52,700 cases formed the basis for subsequent family linkage filtering (see Figure 1 in the main manuscript). After excluding cases with no FDR and no information on mother, 51,778 cases were included in the analysis. *CHD=congenital heart defects, ECA=extracardiac anomalies*

Appendix C: Diagnostic Code Tables

This appendix includes all relevant diagnostic codes used in the study.

Table S1. **ICD codes used to identify CHD cases and relatives with CHD**. For cases, CHD status was defined using ICD-9 and ICD-10 codes. For relatives of cases and controls, CHD status was based on ICD-8, ICD-9, and ICD-10 codes. A full list of all diagnostic codes used in the study is available from the corresponding author upon request.

**ICD-10**	**ICD-9**	**ICD-8**
**Q20.0-Q28.9**	**745A-747X**	**746.09-747.90**

Table S2. ICD codes used to define severe CHD. Based on EUROCAT 2018 and early surgical repair criteria.

	**ICD8**	**ICD9**	**ICD10**
**Codes for Severe CHD**	Q20.0-Q20.5, Q21.2, Q21.3, Q22.0, Q25.5, Q22.4-Q22.6, Q23.0, Q23.4, Q25.1, Q25.2, Q26.2	745A-745D, 745G, 746B-746D, 746H, 747B	746.09, 746.19, 746.29, 746.37, 746.47, 746.64, 746.73, 746.74, 747.19
**Repaired CHD < 1 year of age**	All ICD codes + surgery codes from Chapter F, Section A-H, J-N, Q, X, and W; for surgery 1963-1996 codes: 3xxx		

Table S3. **ICD codes used for exclusion criteria.** Applied to reduce misclassification of physiological or misattributed CHD.

	ICD 10	ICD9	ICD8
**Physiological Conditions and specific exclusions**			
PDA preterm	Q25.0 AND P07	747A AND 765	747.09 AND 777
PFO	Q21.1	745F	746.4
PDA	Q25.0	747A	747.0
PS	Q22.1, Q22.3	746A	746.63
PS branch	Q25.6, Q25.7	747D	747.34, 747.39
Isolated unspecified malformation of the circulation system	Q28.9	747X	747.9
Isolated AVB	Q24.6	746W	746.86
**Misclassification of Fetal CHD in Mother's Records**			
Codes for miscarriage, ToP, IUFD after GA 12 weeks	O02.1, O03, O04 (NOT O04.9A, O04.9B), O05, O06, O07, O36.4	632X, 634, 635, 636, 637, 638, 646D, 656E	634.60, 634.90, 640, 641, 642, 643, 644, 645.93
	**Except for**		
	Q21.0, Q21.1, Q21.9, Q22.1, Q22.2, Q23.1, Q23.3, Q24.3, Q24.4, Q25.0, Q25.3, Q25.6	745E, 745F, 745X, 746A, 746E, 746G, 747A	746.39, 746.42, 746.59, 746.63, 747.09
**Registry Data Validity Concerns-Single entry and specific types severe CHD**			
	Q20.0, Q20.1, Q20.2, Q20.3, Q20.4, Q20.5, Q20.6, Q21.2, Q21.3, Q21.4, Q22.0, Q22.4, Q22.5, Q22.6, Q23.0, Q23.4,Q24.2, Q24.3, Q24.4, Q24.5, Q24.6, Q24.8, Q25.1, Q25.2 , Q25.3, Q25.5, Q25.6, Q25.7, Q26.2, Q26.3, Q26.4	745A, 745B, 745C, 745D, 745G, 745H, 745W, 746B, 746C, 746D, 746H, 747B, 747C, 747D	746.09, 746.19, 746.29, 746.37, 746.43, 746.46, 746.47, 746.54, 746.59, 746.64, 746.73, 746.74, 746.84, 746.86, 747.19, 747.29, 747.34, 747.39, 747.59, 747.69
**Registry Data Validity Concerns-Lethal heart defect**			
TGA NOT in combination with Q20.5 OR (Q22.1 AND Q21.0) OR (746A AND 745E) OR (746.63 AND746.39)	746.19	745B	Q20.3
PA	746.64	746A	Q22.0
HLHS	746.74	746H	Q23.4
TA/HRHS		746B	Q22.4 OR Q22.6
DILV/single ventricle	746.37	745D	Q20.4

PDA, patent ductus arteriosus; PFO, patent foramen ovale; PS, pulmonary stenosis; AVB, atrioventricular block; GA, gestational age; IUFD, intrauterine fetal death; ToP, termination of pregnancy; TGA, transposition of the great arteries; PA, pulmonary atresia; HLHS, hypoplastic left heart syndrome;TA, Tricuspid Valve atresia; HRHS, hypoplastic right heart syndrome; DILV, double inlet left ventricle

Table S4. ICD codes for genetic abnormalities and extra-cardiac malformations. **Used for descriptive and sensitivity analyses.**

	ICD-10	ICD-9	ICD-8
**Genetic abnormalities**	Q87, Q89.7, Q89.8A-C, Q90-Q99	758A-H, 758W, 758X	759.30, 759.40, 759.41, 759.42, 759.48, 759.50<br>759.51, 759.52, 759.53<br>759.59, 759.80-87
**Extra-cardiac malformations**			
	Q00-Q07, Q10-Q18, Q30-Q34, Q35-Q37, Q38-Q45, Q50-Q56, Q60-Q64, Q65-Q79, Q89.1-Q89.2, Q89.3, Q89.4, Q89.9, Q86	740-742, 743-744, 748, 749, 750-751, 752, 753, 754-756, 759B-759C, 759D, 759E, 759H, 759W, 759X	740-743, 744-745, 748, 749, 750-751, 752, 753, 754-756, 758.10-29, 758.38-99, 759.00, 759.10-29, 758.30

Table S5. ICD codes for identification of maternal comorbidities. Includes diabetes, hypertension, and obesity, used as modifiers in interaction analyses. Maternal comorbidities were defined as having at least two records with the relevant ICD codes either in the National Patient Register (NPR) at least one year prior to the delivery date or at the first antenatal visit recorded in the Medical Birth Register (MBR).

	ICD 10	ICD 9	ICD 8
Maternal diabetes	E10-E14	250	250
Maternal Obesitas	E65-E68	278A-276B	277.99
Maternal hypertension	I10-I15	401-405	400-404

Appendix D: CHD Prevalence by Birth Year

To estimate annual CHD prevalence, the number of live births per year between 1987 and 2017 was retrieved from Statistics Sweden (total n = 3,214,974).

In our study population, total CHD prevalence increased markedly from the mid-1990s to early 2010s, followed by a slight decline after 2014, likely reflecting improved detection, diagnostic coding, and register coverage rather than a true increase in disease burden. The introduction of ICD-10 in 1997 allowed more precise classification of congenital heart defects, while the inclusion of outpatient data in the National Patient Register from 2001 enhanced the capture of milder cases. In contrast, the prevalence of severe CHD remained relatively stable throughout the study period.

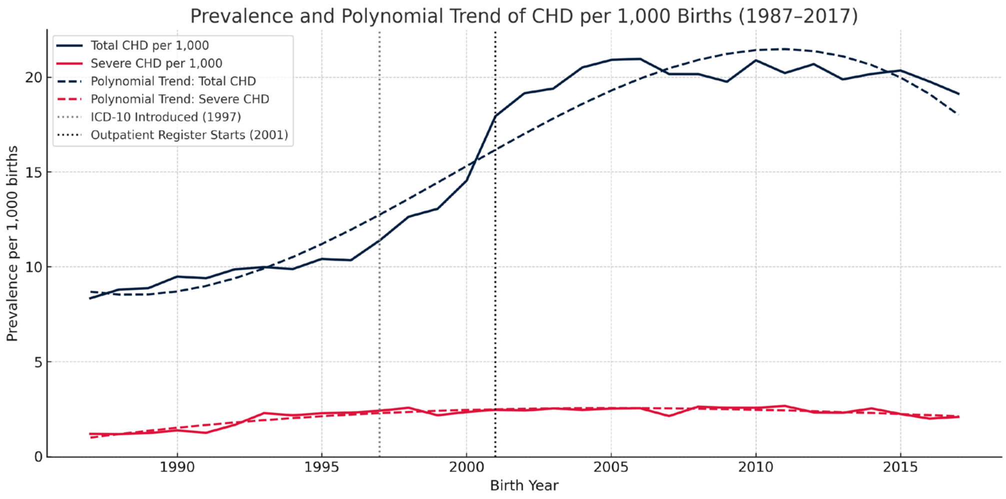

Figure S2. Annual Prevalence of CHD by Birth Year (1987–2017). Prevalence estimates stratified by CHD severity as defined by EUROCAT 2018 and early repair status. Trends in the prevalence of total and severe congenital heart defects (CHD) per 1,000 live births in Sweden from 1987 to 2017. Dashed lines represent third-degree polynomial trendlines.

Appendix E: Frequency of Genetic Syndromes and Extracardiac Anomalies

This section describes the distribution of genetic syndromes and extracardiac anomalies (ECA) among CHD cases. Individuals were categorized as having isolated genetic abnormalities, isolated ECA, or both (Table S6). These categories were used to differentiate syndromic from non-syndromic CHD and to enable stratified and sensitivity analyses.

Table S6. Distribution of Genetic Abnormalities and Extracardiac Malformations Among CHD Cases. This table summarizes the prevalence of documented genetic syndromes and extracardiac anomalies (ECA) among congenital heart disease (CHD) cases.

**Group**	**n**	**% of CHD cases (N = 51,778)**
Neither genetic abnormality nor ECA	39,626	76.5%
Any genetic abnormality or ECA	12,152	23.5%
Genetic abnormality with or without ECA	4,795	9.3%
Isolated genetic abnormality (without ECA)	2,226	4.3%
Isolated ECA (without genetic abnormality)	7,357	14.2%
Both genetic abnormality and ECA	2,569	5.0%

Appendix F: Sensitivity Analyses

Inclusion of Cases Excluded for Registry Data Reliability concerns

To assess the impact of registry-based exclusions on familial associations, we conducted a sensitivity analysis that reintroduced individuals excluded under Criterion 3: Registry Data Reliability (see Appendix B). These exclusions were initially applied to minimize misclassification due to coding inconsistencies, limited follow-up, or implausible clinical profiles. However, they may have inadvertently removed true but milder CHD cases—particularly among subgroups like mothers with more frequent healthcare contact.

In this sensitivity analysis, these cases were retained, while all other criteria and statistical methods remained unchanged. Results were consistent with the primary analysis. (Figure S3). These findings support the robustness of our main results and indicate that exclusion based on registry reliability did not meaningfully bias observed familial patterns.

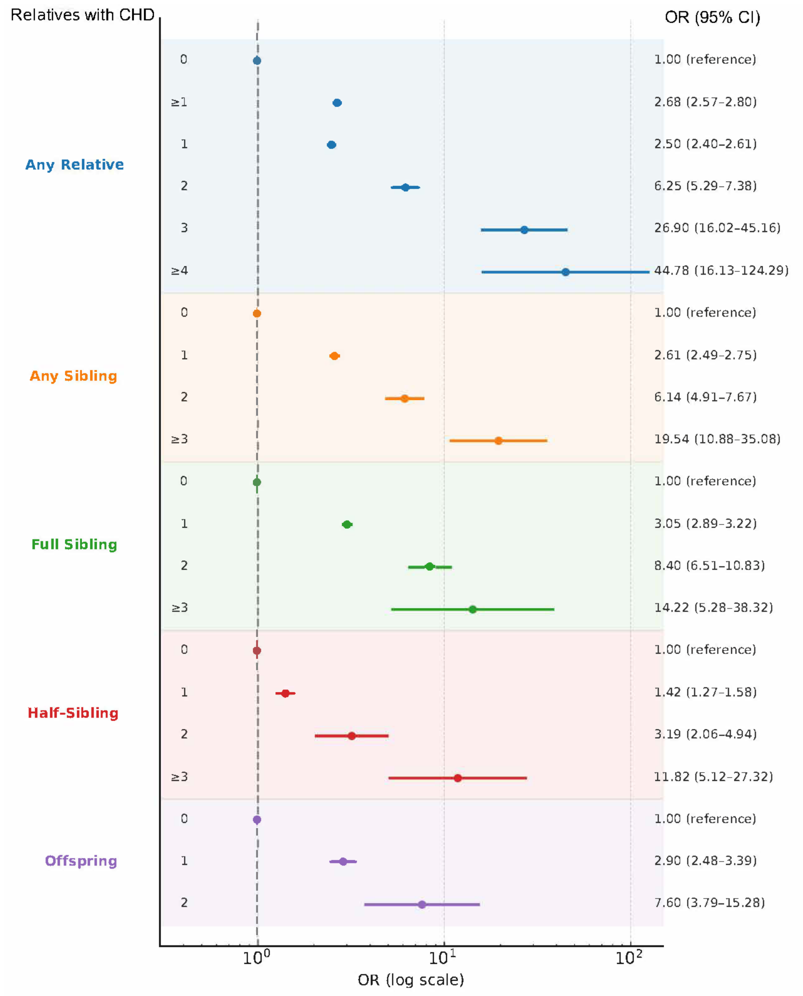

Figure S3. Sensitivity Analysis: Odds Ratios for congenital heart defects by number and kinship of affected relatives (FDR and half-siblings), including cases previously excluded for registry data reliability

Forest plot showing adjusted odds ratios and 95% Confidence Intervals for Congenital Heart Defects by number and kinship of affected relatives, including previously excluded cases due to registry concerns. Logistic regression models adjusted for sex, birth year, county of residence at birth, family size, maternal and paternal age categories, and parental CHD, as appropriate for each kinship type. Robust standard errors clustered by maternal identifier. A log-scale x-axis is used.

Exclusion of index individuals with documented genetic syndromes or extracardiac anomalies

To explore familial recurrence patterns specifically among non-syndromic cases, we performed an analysis restricted to index individuals without documented genetic syndromes or extracardiac malformations. The analysis included 39,626 cases and 401,785 controls with at least one identified relative (FDRs and half-siblings) and identified mother. Dose-response relationship and kinship-specific associations remained consistent (Table S7).

Table S7. Adjusted odds ratios for CHD associated with familial CHD: Sensitivity analysis excluding matched sets with genetic or extracardiac abnormalities

Adjusted odds ratios (ORs) and 95% confidence intervals (CIs) for CHD associated with familial CHD, based on a sensitivity analysis excluding all matched case-control sets where the case had a genetic abnormality or extracardiac malformation. Models were adjusted for birth year, sex, county of residence at birth, family size, and parental age categories where appropriate. Robust standard errors were clustered on maternal ID.

Relatives with CHD	Adjusted OR (95% CI)
≥1 Relative	2.80 (2.68–2.94)
1 Relative	2.62 (2.49–2.75)
2 Relatives	6.39 (5.29–7.72)
3 Relatives	35.50 (19.26–65.44)
≥4 Relatives	36.99 (12.31–111.18)
Per additional Relative with CHD (trend)	2.62 (2.52–2.73)
Mother	3.08 (2.74–3.47)
Father	2.31 (2.03–2.63)
Full sibling	3.31 (3.12–3.51)
Half-sibling (maternal)	1.72 (1.43–2.07)
Half-sibling (paternal)	1.33 (1.15–1.55)
Offspring	2.96 (2.46–3.57)

CHD: congenital heart disease; aOR = adjusted odds ratio; CI = confidence interval;

Interaction Between Familial CHD and Birth Period

We examined whether the association between familial CHD and CHD in the index individual differed by birth period, using an interaction term with a binary cutoff at 2001—the year when outpatient diagnoses were incorporated into the National Patient Register, improving detection of milder CHD.

The familial association was slightly stronger for individuals born in or after 2001 (aOR 2.80, 95% CI 2.66–2.94) than for those born earlier (aOR 2.49, 95% CI 2.32–2.69). On the additive scale, there was a positive interaction (RERI = 1.12, 95% CI 1.03–1.22; P = 0.011), indicating that the combined effect of being born after 2001 and having an affected relative produced more CHD cases than expected from their separate effects. On the multiplicative scale, the ratio of odds ratios (ROR = 0.29, 95% CI 0.06–0.51; P = 0.013) suggested a smaller proportional difference between those with and without familial CHD in later birth cohorts, likely reflecting improved detection of sporadic and milder cases after 2001. Among kinship types, interaction was significant only for maternal half-siblings (RERI = 0.55, P = 0.018; ROR = 1.47, P = 0.020). Interaction analysis was not feasible for offspring due to limited data.

Table S8. Interaction analysis by birth-period

This table presents adjusted odds ratios for CHD in index individuals by birth period (born before 2001 vs 2001 or later), stratified by the presence of affected relatives. Multiplicative interaction was evaluated using the ratio of odds ratios (ROR) with p-values, and additive interaction using the relative excess risk due to interaction (RERI) with 95% confidence intervals. Models were adjusted for sex, birth year (continuous within strata), maternal age, family size, and county of residence, and used robust standard errors clustered by maternal identifier.

Relative with CHD	Birth year (index)	CHD in index yes / no	Adjusted OR (95 % CI) for CHD in relative within strata of birth year
**Any Relative**			
No	Before 2001	13,866/147,534	1.00 (ref)
	2001 or later	33,286/356,559	2.49 (2.32, 2.69)
Yes	Before 2001	1,208/5,171	1.00 (ref)
	2001 or later	3,418 /13,279	2.80 (2.66, 2.94)
Additive scale: RERI = 1.12 (1.03 to 1.22); P = 0.011			
Multiplicative scale: Ratio of ORs = 0.29 (0.06 – 0.51); P = 0.013			
**Mother**			
No	Before 2001	14,923 / 152,277	1.00 (ref)
	2001 or later	36,316 / 368,510	3.60 (2.95, 4.39)
Yes	Before 2001	151 / 428	1.00 (ref)
	2001 or later	388 / 1,328	2.96 (2.62, 3.35)
Additive scale: RERI = –0.62(–1.41 to 0.18); P = 0.127			
Multiplicative scale: Ratio of ORs = 0.82 (0.65 – 1.04); P = 0.099			
**Father**			
No	Before 2001	14,944 / 152,149	1.00 (ref)
	2001 or later	36,421 / 368,534	2.39 (1.95, 2.94)
Yes	Before 2001	130 / 556	1.00 (ref)
	2001 or later	283 / 1,304	2.19 (1.91, 2.50)
Additive scale: RERI = -0.19 (-0.76, 0.38); P = 0.512			
Multiplicative scale: Ratio of ORs = 0.92 (0.72 – 1.17); P = 0.476			
**Full sibling**			
No	Before 2001	14,388 / 150,500	1.00 (ref)
	2001 or later	34,184 / 361,467	3.24 (2.91, 3.61)
Yes	Before 2001	686 / 2,205	1.00 (ref)
	2001 or later	2,520 / 8,371	3.22 (3.03, 3.42)
Additive scale: RERI = -0.00 (-0.39, 0.39); P = 0.980			
Multiplicative scale: Ratio of ORs = 0.99 (0.88 – 1.12); P = 0.911			
**Half sibling (maternal)**			
No	Before 2001	15,005/152,166	1.00 (ref)
	2001 or later	36,481 / 368,684	1.26 (0.94, 1.70)
Yes	Before 2001	69 / 539	1.00 (ref)
	2001 or later	223 / 1,154	1.86 (1.53, 2.25)
Additive scale: RERI = 0.55 (0.09, 1.01); P = 0.018			
Multiplicative scale: Ratio of ORs = 1.47 (1.06 – 2.03); P = 0.020			
**Half sibling (paternal)**			
No	Before 2001	15,000 / 152,066	1.00 (ref)
	2001 or later	36,511 / 368,490	1.13 (0.88, 1.45)
Yes	Before 2001	74 / 639	1.00 (ref)
	2001 or later	193 / 1,348	1.41 (1.20, 1.65)
Additive scale: RERI = 0.27 (-0.08, 0.62); P = 0.137			
Multiplicative scale: Ratio of ORs = 1.25 (0.93 – 1.67); P = 0.141			

CHD: congenital heart disease, aOR = adjusted odds ratio; CI = confidence interval; RERI = relative excess risk due to interaction; ROR = ratio of odds ratios.

Analysis restricted to severe CHD

To explore whether familial recurrence patterns differed by CHD severity, we performed a sensitivity analysis restricted to individuals with severe CHD, defined using EUROCAT 2018 criteria. This subgroup included 7,229 cases and 72,024 matched controls (9.1% of the total study population). The dose–response trend remained consistent, the associations for full siblings and offspring were similar, while the OR for maternal CHD was slightly higher. Associations for half-siblings—especially paternal—were attenuated (Table S9).

Table S9. Adjusted odds ratios for CHD by familial recurrence in severe CHD

Comparison of adjusted odds ratios (ORs) for congenital heart disease (CHD) associated with familial recurrence patterns in the full cohort and among severe CHD cases only. Estimates are adjusted for sex, birth year, county, and family size. Kinship-specific models were further adjusted for parental age and parental CHD where relevant. ORs are based on logistic regression with robust standard errors clustered by maternal identifier.

Relatives with CHD	Adjusted OR (95% CI) Full Cohort	Adjusted OR (95% CI) Severe CHD Only
≥1 Relative	2.71 (2.60–2.83)	2.68 (2.43–2.95)
1 Relative	2.52 (2.41–2.63)	2.53 (2.29–2.79)
2 Relatives	6.42 (5.41–7.63)	6.35 (4.27–9.44)
3 Relatives	29.40 (17.21–50.23)	10.6 (3.04–36.9)
Dose–response trend (per relative)	2.55 (2.46–2.64)	2.53 (2.33–2.75)
Mother	3.12 (2.80–3.47)	3.54 (2.73–4.60)
Father	2.25 (2.01–2.52)	2.38 (1.83–3.11)
Full sibling with CHD	3.22 (3.05–3.40)	3.22 (2.86–3.63)
Half-sibling (maternal)	1.67 (1.40–1.99)	1.48 (1.02–2.16)
Half-sibling (paternal)	1.32 (1.16–1.51)	1.22 (0.85–1.74)
Offspring with CHD	3.16 (2.70–3.70)	3.09 (1.99–4.79)

FDR: First-degree relative, CHD: congenital heart disease

Analysis restricted to singleton births

We conducted a sensitivity analysis excluding twins from both cases and controls. This exclusion resulted in the removal of 16,207 individuals, yielding a study population of 558,114 singleton births (91.2% non-cases and 8.8% cases). Findings were similar to the main analysis (Table S10).

Table S10. Adjusted Odds Ratios (ORs) for CHD by Number of Affected Relatives in Singletons. Adjusted odds ratios (ORs) with 95% confidence intervals (CIs) for congenital heart disease in index individuals by the number of affected relatives (FDRs and half-siblings), based on a sensitivity analysis excluding twins from both cases and controls. Models are adjusted for birth year, sex, county, and family size, with robust standard errors clustered by maternal ID. The trend reflects the per-relative increase in odds of CHD.

Number of Affected Relatives	Adjusted OR	95% CI
0	Reference	Reference
≥1	2.47	2.39–2.56
1	2.29	2.18–2.40
2	5.94	4.97–7.10
3	27.56	16.09–47.21
≥4	45.52	16.43–126.10
Trend (per additional Relative)	2.36	2.27–2.45

FDR: First-degree relative, CHD: congenital heart disease

Further technical specifications and assumptions are available upon request.
